# Identification of Conserved and Diverse Metabolic Shifts during Rice Grain Development

**DOI:** 10.1038/srep20942

**Published:** 2016-02-10

**Authors:** Chaoyang Hu, Takayuki Tohge, Shen-An Chan, Yue Song, Jun Rao, Bo Cui, Hong Lin, Lei Wang, Alisdair R. Fernie, Dabing Zhang, Jianxin Shi

**Affiliations:** 1 Joint International Research Laboratory of Metabolic & Developmental Sciences, SJTU-University of Adelaide Joint Centre for Agriculture and Health, School of Life Sciences and Biotechnology, Shanghai Jiao Tong University, 800 Dongchuan Road, Minghan District, Shanghai 200240, China; 2 Central Metabolism Group, Max Planck Institute of Molecular Plant Physiology, 14476 Potsdam-Golm, Germany; 3 Agilent Technology, Inc. Taipei, Taiwan; 4 Agilent Technology, Inc. Shanghai, China; 5 Jiangxi Cancer Hospital, No. 519 East Beijing Road, Nanchang 330029, China; 6 Agilent Technology, Inc. Beijing 100000, China; 7 Shanghai Ruifeng Agro-biotechnology Co., Ltd, Room 108, No 233 Rushan Rd., Shanghai 200120, China

## Abstract

Seed development dedicates to reserve synthesis and accumulation and uncovering its genetic and biochemical mechanisms has been a major research focus. Although proteomic and transcriptomic analyses revealed dynamic changes of genes and enzymes involved, the information regarding concomitant metabolic changes is missing. Here we investigated the dynamic metabolic changes along the rice grain development of two *japonica* and two *indica* cultivars using non-targeted metabolomics approach, in which we successfully identified 214 metabolites. Statistical analyses revealed both cultivar and developmental stage dependent metabolic changes in rice grains. Generally, the stage specific metabolic kinetics corresponded well to the physiological status of the developing grains, and metabolic changes in developing rice grain are similar to those of dicot Arabidopsis and tomato at reserve accumulation stage but are different from those of dicots at seed desiccation stage. The remarkable difference in metabolite abundances between *japonica* and *indica* rice grain was observed at the reserve accumulation stage. Metabolite-metabolite correlation analysis uncovered potential new pathways for several metabolites. Taken together, this study uncovered both conserved and diverse development associated metabolic kinetics of rice grains, which facilitates further study to explore fundamental questions regarding the evolution of seed metabolic capabilities as well as their potential applications in crop improvement.

Rice (*Oryza sativa*) serves not only as an important staple food source feeding half of the world’s population but also as an excellent model for plant research. Understanding of the molecular mechanism underlying rice seed development, an important process dedicated to provide seeds for food and nutrition and an essential component of rice life cycle, is of great interest among multiple field scientists. Rice seed development is a complex and coordinated process, which is coupled with many genetic, physiological, metabolic, and signaling pathways, and affected by both internal and external stimuli^1^,^2^,^3^,^4^.

Generally, rice seed development includes two major phases (embryo and endosperm development) including separate but overlapping stages of embryogenesis, maturation, desiccation and dormancy^1^,^3^,^5^,^6^. The coordinated process of rice seed development relies on different sets of genes/proteins expressed at the right time. Previous studies on transcriptomic and proteomic changes at different developmental stages, especially at the reserve accumulation stage, have largely broadened our understanding of the molecular regulation and metabolic networks of rice seed development^3^,^5^,^7^,^8^,^9^,^10^,^11^,^12^. Transcriptomic analysis revealed that levels and types of expressed genes vary greatly among different developmental stages, and it is assumed that the expression levels of many genes increased or decreased before or corresponding to profound physiological and morphological changes in rice grains^3^,^5^,^11^. It is also found that there are detectable inter-cultivars differences in the expressed genes in developing rice grains^3^,^5^,^7^,^11^. Proteomic analysis studies revealed similar developmental stage dependent expression patterns (levels and types) of proteins along the grain development^6^,^8^,^12^.

Rice seed development involves the synthesis, interconversion, and accumulation of numerous metabolites to form and accumulate various macromolecules, serving as the building blocks of the essential parts of the seed and the intrinsic nutritional and reserve resources. Although abovementioned transcriptomic and proteomic analyses have revealed the roles of genes and proteins in rice seed development, the simultaneously occurred metabolomics changes, representing the end products of genomic, transcriptomic, and proteomic regulatory processes, could not be simply deduced from those data. The flow of information deriving from genes is in reality networked by many loops with their downstream products, resulting in a complex and dynamic system of transcripts, proteins and metabolites. As a consequence, changes in the quantities of individual gene or enzyme might have little effect on metabolic fluxes through pathways, but may have significant effects on the concentrations of individual metabolites^13^,^14^. Thus, a detailed analysis of the metabolome is a representation of the phenotype^15^. Although metabolomics has been applied to study rice biology such as metabolic variation^16^,^17^,^18^,^19^,^20^,^21^ and genetically modified rice^16^, it has never been employed to study rice seed development, an important process that is of significance not only in fundamental research for rice development biology but also in applied research for rice breeding.

Previous metabolomics studies in Arabidopsis seed development revealed that seed maturation is accompanied with a significant reduction of most detected metabolites to guarantee an efficient accumulation of reserves, and that seed desiccation is associated with a striking metabolic shift to accumulate distinct metabolites^22^. Metabolomics study in tomato revealed similarities and differences between flesh and seeds during fruit development, and also pointed that most of the detected metabolites decline as fruit developed^23^. Both studies provided new insights into the relationship between seed development and metabolic changes, and laid foundations for potential application in crop improvement. Because the endosperm accounts for the majority of the rice seed, while the embryo constitutes the major proportion of the seed of Arabidopsis or tomato, we are interested to know if the metabolic kinetics of seed development in monocot rice differs from that in dicot Arabidopsis or tomato. We found previously that mature seeds of *Japonica* and *indica* rice differ profoundly in their metabolite contents and networks^20^, however, we did not know when this metabolic difference is formed.

In this study, non-targeted metabolomics was employed to uncover the kinetic metabolic changes along the grain development in two *japonica* and two *indica* cultivars. This study revealed inter-subspecies and intra-subspecies metabolic differences in developing grain in addition to conserved developmental stage dependent metabolic dynamics. These results are discussed in terms of general models of seed development and with regard to rice grain improvement.

## Results

### Kinetic patterns of developing rice grain metabolomes

**Figure 1:**
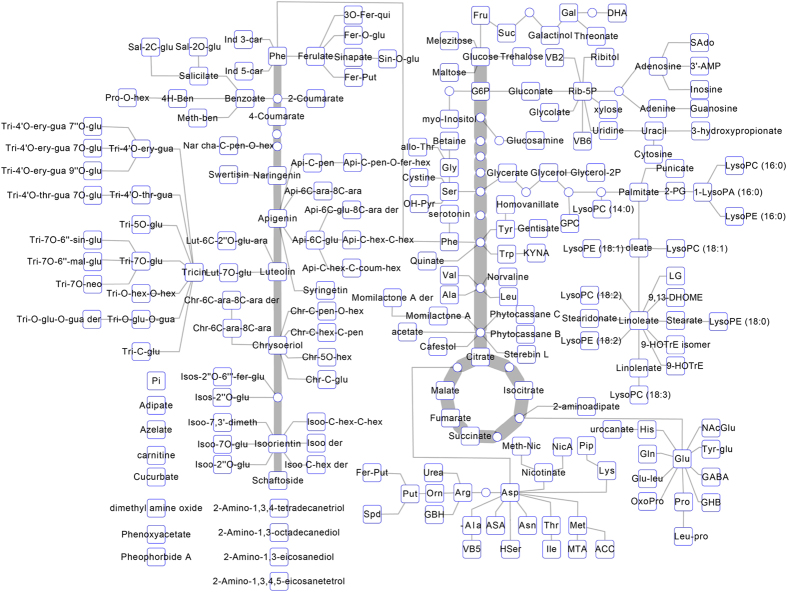
A simplified metabolic map of rice grain based on metabolites identified by GC-MS and UHPLC-MS in this study. Squares and circles denote metabolites detected and undetected in this study, respectively. Full metabolite names refer to Supplementary Table S1.

In order to investigate the metabolic kinetics of rice grains along rice seed development, samples of grains at 7, 10, 14, 28 and 42 days after flowering (DAF) from two *indica* cultivars (Qingfengai and 9311) and two *japonica* cultivars (Nipponbare and Nongken 58) were collected and subjected to metabolic profiling analysis based on gas chromatography (GC)-mass spectrometry (MS) and ultra-high performance liquid chromatography (UHPLC)-MS. Among a total of 3011 intensive signals, the exact chemical structure of 214 metabolites were identified (Fig. 1 and Supplementary Table S1), including 44 amino acids and dipeptides, 41 carbohydrates and organic acids, 11 cofactors, 11 nucleotides, 33 lipids and 69 secondary metabolites (including flavonoids, hydroxycinnamate derivatives, phenylpropanoids, terpenes and diterpenoid phytoalexins) and five other compounds.

**Figure 2:**
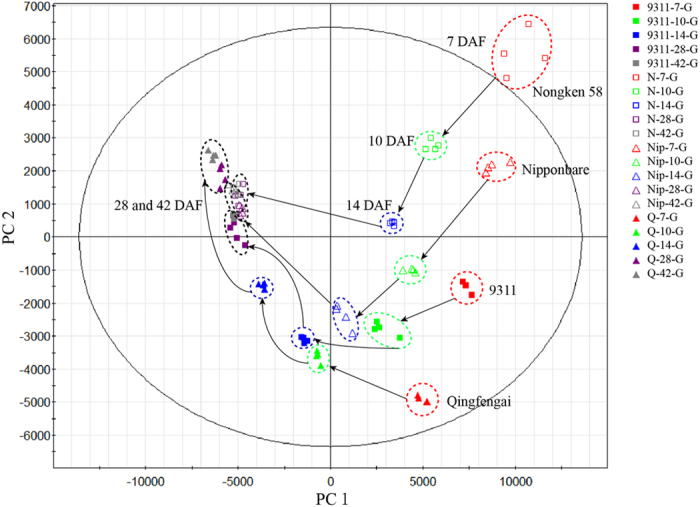
Principal component (PC) analysis of the metabolomes of developing rice grain. Red, green, blue, violet and gray colors represent samples at 7, 10, 14, 28 and 42 DAF, respectively. Box, square, open triangle and triangle denote grain metabolomes of 9311, Nongken 58, Nipponbare and Qingfengai, respectively. PC 1 explains 53.3% of variance distinguishing rice grains from different developmental stages.

Principle component analysis (PCA) on the 3011 metabolic features was subsequently performed to view the kinetic metabolome patterns of the developing rice grains. Notably, developing rice grains of two *japonica* cultivars and two *indica* cultivars showed a similar dynamic pattern in the changes of their metabolomes (Fig. 2). During early stages till 14 DAF, the separation among developing rice grain samples at different stages of the same cultivar or among those of different cultivars at the same stage was clear and wide, revealing metabolomes dependent on both developmental stages and cultivars (Fig. 2). However, during later stages from 28 DAF to 42 DAF, the separation between samples of 28 DAF and those of 42 DAF was almost indistinguishable, indicating the difference of rice grain metabolome at 28 and 42 DAF were much smaller compared to those at 7, 10 and 14 DAF. Therefore, along the development, the PCA plots of samples from four cultivars at the same stage became closer and closer, indicating that as the seed develops, the metabolic variation among grains from different cultivars is getting smaller and smaller.

**Figure 3:**
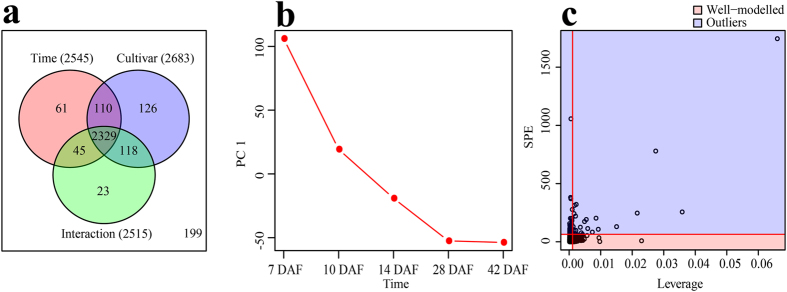
Result of two-way ANOVA and ASCA. (**a**) Venn diagram summary of results from two-way ANOVA. (**b**) Major pattern associated with Time (developmental stage) (**c**) ASCA selection of important variables associated with Time (developmental stage) by Leverage/SPE analysis. These analysis was performed in MetATT website (http://metatt.metabolomics.ca/MetATT/).

Because PCA is unable to reveal which factor causes the observed variation, two-way ANOVA (Analysis of Variance) was then conducted to decompose the raw data to further dissect developmental stage, cultivar or their interaction derived variations. The abundances of 2545, 2683 and 2515 metabolic features were significantly affected by developmental stage, cultivar and their interaction, respectively, among them, the abundances of 2329 metabolic features were simultaneously affected by developmental stage, cultivar and their interaction (Fig. 3a and Supplementary Table S2). In addition, ANOVA-Simultaneous Component Analysis (ASCA), a multivariate extension of univariate ANOVA approach^24^, was performed to provide an interpretation of each experimental factor and to identify the major patterns associated with each factor. ASCA result revealed that 40.4%, 24.8% and 20.9% of observed metabolic variations could be explained by developmental stage, cultivar and their interaction, respectively (Supplementary Fig. S1). Developmental stage score plots based on PC1 of the corresponding submodels showed that the scores gradually decreased along the time (Fig. 3b), which is consistent with PCA result shown in Fig 2, in which metabolomes of different cultivars shifted to the same direction along development. Cultivar score plots showed that different cultivars differ in their PC1 scores; Nongken 58 and Qingfengai had the highest and the lowest score, respectively (Supplementary Fig. S2a). Interaction score plot of Qingfengai decreased while that of Nongken 58 increased and those of Niponbare and 9311 were relative stable, along seed development (Supplementary Fig. S2b). The difference of the interaction scores of each cultivar at 7 DAF was the highest and those at 10 DAF and 14 DAF were quite small (Supplementary Fig. S2b). Leverage/squared prediction error (SPE) plots were made to correlate the metabolic features with the experimental factors^25^. Leverage evaluates the importance of the metabolite to the model, and SPE tests the fitness of the model for particular metabolites. Metabolites with high leverage and low SPE that contribute significantly to the model were picked out as well-modeled metabolites. By doing so, a total of 153 metabolic features stood out based on the major pattern of developmental stage, 12 of which were structurally identified (Fig. 3c and Supplementary Table S3), and 9 out of them showed similar dynamic patterns as shown in Supplementary Fig. S4, which was in accordance with the score plot result shown in Fig. 3b. In addition, a total of 56 and 143 metabolic features stood up based on the major patterns of cultivar and interaction, respectively (Supplementary Fig. S3 and Supplementary Table S3).

So far, we analyzed only the global metabolic change patterns of developing rice grain with detected 3011 metabolic features. Now, we started to investigate into the metabolic changes occurred at each developmental stage with 214 identified metabolites.

### Metabolic changes of rice grain during reserve accumulation

**Figure 4:**
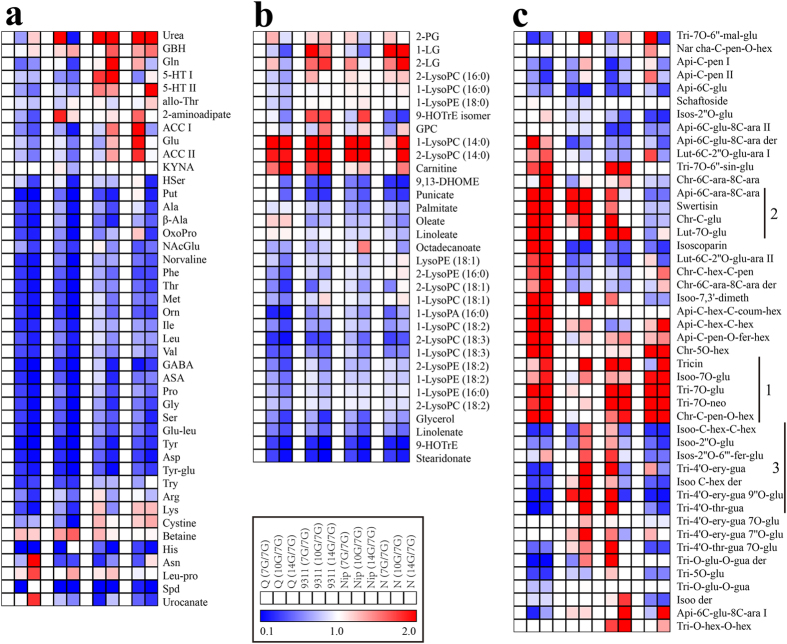
Heat map of metabolite changes in rice grains at reserve accumulation stage (7, 10 and 14 DAF). (**a**) amino acids and dipeptides. (**b**) lipids. (**c**) flavonoids. Q, Nip and N are shorted for three rice cultivars, Qingfengai, Nipponbare and Nongken 58, respectively. Ratios of fold changes are given by shades of red or blue colors according to the scale bar. Data represent mean values of four biological replicates for each cultivar and time point. Statistical analysis was performed using t-test (Supplementary Table S4). For full metabolite names, refer to Supplementary Table S1.

To uncover the metabolic alterations in developing grains during the period of reserve accumulation, the levels of the 214 identified metabolites at 10 DAF and 14 DAF were compared with those at 7 DAF of the same cultivar to eliminate the cultivar-dependent variation. As reserve accumulation progressed, levels of 69.2% (27 out of 39) of amino acids and their derivatives decreased dramatically from the highest level at 7 DAF to the lowest level at 14 DAF in all four cultivars albeit to varying degree (Fig. 4a and Supplementary Table S4). For example, levels of histidine and spermidine decreased more than 10 times while *N*-acetylglutamate and tryptophan decreased about three times at 14 DAF compared with those at 7 DAF. In striking contrast, levels of two amino acids, betaine and γ-guanidinobutyric acid (GBH), and one dipeptide (Leu-pro), increased significantly at 10 and 14 DAF as compared with those at 7 DAF. Most of carbohydrates and organic acids showed the similar change pattern to those of amino acids (Supplementary Fig. S5a). However, the level of sucrose was rather constant in all four cultivars, indicating a sufficient carbon supply from the leaf during this period. The similar decreases in the contents of cofactors and nucleotides were also observed in all four cultivars although it is important to note that the extraction protocol used here is not ideal for conserving the nucleotides in their *in vivo* state so these specific data need to be interpreted with caution (Supplementary Fig. S5b, 5c). Although the levels of many lipids also decreased during reserve accumulation (Fig. 4b), the degree of the decline in lipids was smaller than aforementioned metabolites. Among annotated secondary metabolites, levels of most detected hydroxycinnamate derivatives decreased dramatically during reserve accumulation especially at 14 DAF in all four cultivars (Supplementary Fig. S5d), while the level of methylbenzoate (Meth-ben) increased significantly at 14 DAF (Supplementary Fig. S5e). Among detected flavonoids, the levels of only two flavonoids, isoorientin C-hexoside-C-hexoside I (Isoo-C-hex-C-hex I) and apigenin-6-C-glucoside (Api-6C-glu), significantly decreased while levels of five flavonoids, such as tricin and isoorientin 7-O-glucoside (Isoo-7O-glu) increased at 10 DAF and 14 DAF in all four cultivars (Fig. 4c, label 1).

A few of the primary metabolites, however,showed a cultivar specific change manner. For example, glutamine and gluamate levels increased in two *japonica* cultivars (10 DAF in Nongken 58 and 14 DAF in Nipponbare) but decreased in two tested *indica* cultivars after 7 DAF; the level of 5-methylthioadenosine (MTA), a nucleotide, ascended at 10 DAF and 14 DAF then decreased dramatically in the two *japonica* cultivars while it decreased gradually from 7 DAF across seed development in the two *indica* cultivars. Levels of flavonoids exhibited the most striking cultivar specific changes (Fig. 4c). For example, levels of five flavonoids including chrysoeriol C-glucoside (Chr-C-glu), swertisin and isoorientin 7,3′-dimethyl ether (Isoo-7,3′-dimeth) increased in the two *indica* cultivars but decreased in Nongken 58 (Fig. 4c, label 2); Levels of isoscoparin 2”-O-(6”‘-(E)-feruloyl)-glucopyranoside (Isos-2“O-6”‘-fer-glu) and tricin 4′-O-(erythro-β-guaiacylglyceryl) ether (Tri-4′O-ery-gua), tended to increase in 9311 and Nipponbare but decrease in Qingfengai and Nongken 58 (Fig. 4c, label 3).

### Metabolic changes of rice grain during seed desiccation

**Figure 5:**
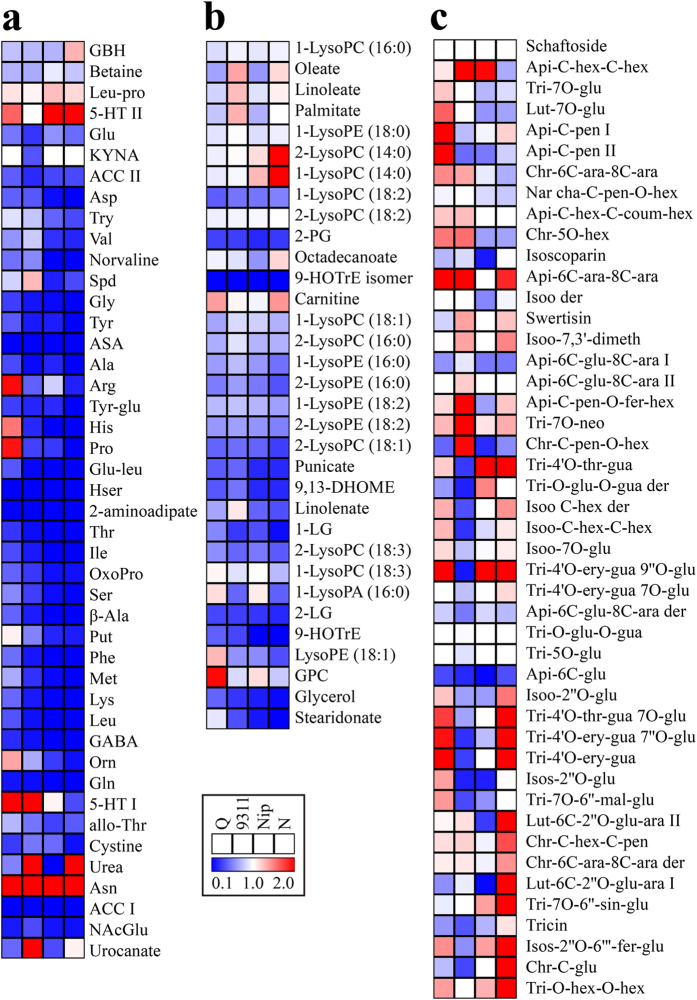
Heat map of metabolite changes in rice grains at desiccation stage (28 DAF vs 14 DAF). (**a**) amino acids and dipeptides. (**b**) lipids. (**c**) flavonoids. Q, Nip and N are shorted for three rice cultivars, Qingfengai, Nipponbare and Nongken 58, respectively. Ratios of fold changes are given by shades of red or blue colors according to the scale bar. Data represent mean values of four biological replicates for each cultivar and time point. Statistical analysis was performed using t-test (Supplementary Table S5). For full metabolite names, refer to Supplementary Table S1.

We also compared the metabolite levels between samples of 28 DAF and 14 DAF to explore the metabolic shift during the transition from reserve accumulation to seed desiccation. Levels of 102, 125, 125 and 137 metabolites declined further while those of 23, 14, 12 and 12 metabolites increased during this transition in Qingfengai, 9311, Nipponbare and Nongken 58, respectively (Supplementary Table S5). Levels of 57.8% (26 out of 45) of amino acids and their derivatives decreased significantly with varying degrees dependent on cultivar and metabolite itself (Fig. 5a and Supplementary Table S5). For example, levels of argininosuccinate (ASA) and homoserine (Hser) decreased more than 25 times while glutamate (Glu) decreased only two to three times in all four cultivars, and that of glycine (Gly) decreased 20 times in Nongken 58 but only 3 times in Qingfengai. Notably, the level of asparagine (Asn) increased in all four cultivars. Levels of 34.1% (14 out of 41) of carbohydrates and organic acids, such as malate, succinate, fumarate, galactinol, ribitol and *myo*-inositol, also dramatically decreased in all four cultivars (Supplementary Fig. S6a and Supplementary Table S5). Though sucrose increased in Qingfengai and Nipponbare and decreased in the other two cultivars during seed desiccation, the degrees of the change were less than 33%, much smaller than the changes of other carbohydrates. Additionally, the change of five carbohydrates and organic acids, such as trehalose and 4-hydroxybutyric acid (GHB) were not statistically significant in all four cultivars (Supplementary Table S5). Levels of nearly all cofactors and nucleotides decreased dramatically (Supplementary Fig. S6b, S6c) and those of 51.5% (17 out of 33) of the lipid species measured decreased significantly in at least three cultivars (Fig. 5b and Supplementary Table S5).

Levels of 40% (six out of 15) of the hydroxycinnamate derivatives decreased dramatically in at least three cultivars during seed desiccation (Supplementary Fig. S6d and Supplementary Table S5). For example, N-feruloylputrescine (Fer-Put I and Fer-Put II) and 3-O-feruloylquinic acid (3O-Fer-qui) decreased more than 15 fold. Again, methylbenzoate contents increased but 4-hydroxybenzoic acid decreased greatly at this stage (Supplementary Fig. S6e). Unlike other metabolites, the level of only one flavonoid (apigenin-6-C-glucoside, Api-6C-glu) decreased while levels of 54.3% (25 out of 46) flavonoids were not significantly changed in all four cultivars (Fig. 5c and Supplementary Table S5).

The change of many metabolites during seed desiccation also showed cultivar specific patterns. Levels of arginine (Arg) and proline (Pro) increased about two times in Qingfengai but decreased significantly in other three cultivars especially in Nongken 58. Levels of two phospholipids, 2-LysoPC (14:0) and 1-LysoPC (14:0), increased significantly only in the two *japonica* cultivars. Levels of hydroxycinnamate derivatives, including 1-O-sinapoyl-β-D-glucose (Sin-O-glu), 1-O-Feruloyl-β-D-glucose I (Fer-O-glu I), salicylic acid 2-C-β-D-glucopyranoside (Sal-2C-glu), and Pro-O-hex I, decreased sharply in Qingfengai, 9311 and Nongken 58 but not in Nipponbare. The content of two flavonoids changed significantly in three cultivars, i.e. isoscoparin decreased in Qingfengai, 9311 and Nipponbare, while tricin 7-O-neohesperidoside (Tri-7O-neo) increased in 9311, Nipponbare and Nongken 58. Similarly changes in terpenes and diterpenoid phytoalexins were also cultivar specific (Supplementary Fig. S6f). Cafestol and phytocassane C increased 37.8 and 55.5 times in Qingfengai, to a much lesser degree (3.2 and 2.5 times) in 9311, but decreased more than three-fold in Nongken 58 and were invariant in Nipponbare.

### Metabolic changes of rice grain during dormancy

We further compared the metabolomes of rice grains at 42 DAF with those at 28 DAF to investigate metabolic changes occurred at the stage of dormancy. Rice grain samples at 42 DAF were fairly separated from those at 28 DAF in all four cultivars as shown in PCA chart (Supplementary Fig. S7), which indicated that the metabolome of rice grain kept changing even after desiccation. However, the degrees of the metabolic changes at this stage were somewhat smaller than those of reserve accumulation stage but much smaller than those of desiccation stage.

**Figure 6:**
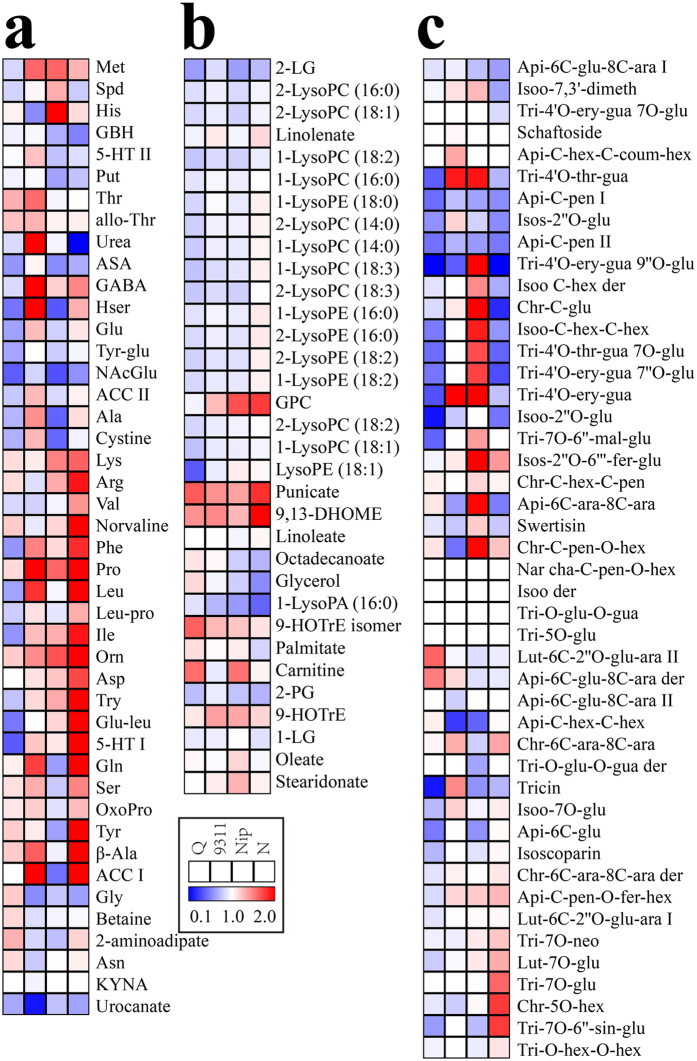
Heat map of metabolite changes in rice grains at dormancy stage (42 DAF vs 28 DAF). (**a**) amino acids and dipeptides. (**b**) lipids. (**c**) flavonoids. Q, Nip and N are shorted for three rice cultivars, Qingfengai, Nipponbare and Nongken 58, respectively. Ratios of fold changes are given by shades of red or blue colors according to the scale bar. Data represent mean values of four biological replicates for each cultivar and time point. Statistical analysis was performed using t-test (Supplementary Table S6). For full metabolite names, refer to Supplementary Table S1.

Levels of 14, 18, 17 and 26 amino acids and dipeptides fluctuated significantly in Qingfengai, 9311, Nipponbare and Nongken 58, respectively, but none of them changed consistently in all four cultivars (Supplementary Table S6 and Fig. 6a). For example, methione (Met), phenylalanine (Phe) and proline (Pro) increased in 9311, Nipponbare and Nongken 58 but not in Qingfengai. Glutamine (Gln) and tyrosine (Tyr) increased in 9311 and Nongken 58, but decreased in Nipponbare. Levels of 11, four, 16 and 14 carbohydrates and organic acids changed significantly in Qingfengai, 9311, Nipponbare and Nongken 58, respectively (Supplementary Table S6 and Supplementary Fig. S8a). Similar to amino acids, none of the carbohydrates changed significantly in all four cultivars. Pantothenic acid (VB5) was the only cofactor whose level decreased in all four cultivars, and no cofactors showed significantly increased levels (Supplementary Fig. S8b). Levels of five, two, ten and eight nucleotides changed significantly in Qingfengai, 9311, Nipponbare and Nongken 58, respectively. (Supplementary Fig. S8c). Adenine was the only nucleotide whose level decreased in all four cultivars, and the changes of the other nucleotides were cultivar specific. Levels of two free fatty acids, punicate and 13-HODE + 9-HODE, increased in all four cultivars (Fig. 8b). Similarly, the levels of three lipids, 2-palmitoylglycerol (2-PG), 2-linoleoylglycerol (2-LG) and 2-LysoPC (18:3), decreased, while Sn-glycero-3-phosphocholine (GPC) increased in three cultivars.

Levels of six to seven hydroxycinnamate derivatives changed significantly in each cultivar but none of them changed simultaneously (Supplementary Fig. S8d). For example, levels of 3-O-feruloylquinic acid (3O-Fer-qui) and 1-O-feruloyl-β-D-glucose II (Fer-O-glu II) increased significantly in 9311 but decreased in Qingfengai and Nipponbare. One of the phenylpropanoids, 4-hydroxybenzoic acid, decreased significantly in all four cultivars while the other one, methylbenzoate, increased in 9311 and Nipponbare (Supplementary Fig. S8e). Levels of five, two, five and eight flavonoids changed significantly in Qingfengai, 9311, Nipponbare and Nongken 58, respectively, but none of them changed simultaneously in all four cultivars (Fig. 6c). Levels of three diterpenoid phytoalexins (phytocassane C, momilactone A and momilactone A derivative) and one terpene (cafestol) decreased significantly only in Qingfengai but not others (Supplementary Fig. S8f).

### Metabolic differences between two *japonica* and two *indica* rice grains

Mature rice seeds significantly differ in their abundances and networks of metabolites between *japonica* and *indica* subspecies^20^, however, little is known whether it holds true at all developmental stages. To address this question, a heatmap of metabolite ratios (hereafter ratio indicates the mean value of two *japonica* divided by the mean value of two *indica*) between two *japonica* cultivars (Nipponbare and Nongken 58) and two *indica* cultivars (Qingfengai and 9311) at different developmental stages was constructed (Supplementary Fig. S9 and Supplementary Table S7).

The results showed that the difference in metabolite abundance between *Japonica* cultivars and *indica* cultivars was developmental stage dependent (Supplementary Fig. S9), because *Japonica* cultivars and *indica* cultivars differed in the extent of their declines in the abundances of most of the detected metabolites (Supplementary Table S4 to S6). For example, levels of 17 amino acids, 19 carbohydrates and organic acids, four cofactor, three nucleotides, and 15 lipids were significantly higher in the two *japonica* cultivars at 7 DAF, the ratios of them peaked at 10 DAF or 14 DAF, and sharply declined afterwards (Supplementary Table S7). In addition, levels of 14 amino acids, five carbohydrates and organic acids, five cofactor, six nucleotides, and 13 lipids were significantly lower in the two *japonica* cultivars at 7 DAF, and then fluctuated afterwards (Supplementary Table S7). Some primary metabolites were constitutively higher or lower in *japonica* than in *indica* at all developmental stages. For instance, levels of GBH, indole 5-carboxylic acid, VB5, cytosine II, and carnitine were constitutively lower while those of GABA, succinate, trigonelline, adenine, and LysoPC (18:1) were constitutively higher in *japonica* cultivars at all developmental stages (Supplementary Fig. S9). In addition, levels of dozens of metabolites (such as, histidine, spermidine, myo-inositol, dehydroascorbate, and succinyladenosine) did not differ at 7 DAF, but differed significantly at 10 and 14 DAF (Supplementary Table S7).

As for secondary metabolites, levels of six hydroxycinnamate derivatives, 13 flavonoids, two phenylpropanoids, and two diterpenoid phytoalexins were higher in *japonica* at 7 DAF, and the ratios peaked at 10 DAF, and then fluctuated, either kept constitutively high or reduced (Supplementary Table S7). On the other hand, levels of five hydroxycinnamate derivatives, six flavonoids, and one diterpenoid phytoalexin were lower in *japonica* at 7 DAF, and fluctuated afterwards (Supplementary Table S7). Levels of two hydroxycinnamate derivatives (4-coumarate, protocatechuic acid O-hexoside II), one phenylpropanoid (methylbenzoate), and five flavonoids (Apigenin-6-C-β-glucoside-8-C-α-arabinoside derivative, tricin, tricin 7-O-neohesperidoside, tricin 7-O-β-D-glucopyranoside, and tricin 7-O-β-D-glucopyranoside) were consistently higher while those of one hydroxycinnamate derivative (N-Feruloylputrescine II) and four flavonoids (apigenin 6-C-α-L-arabinosyl-8-C-β-L-arabinoside, chrysoeriol C-glucoside, C-pentosyl-apeignin O-feruloylhexoside, isoorientin 7,3′-dimethyl ether) in *japonica* at all developmental stages (Supplementary Fig. S9g – S9i).

### Metabolite-metabolite correlations in developing grains

Since all tested four cultivars showed similar kinetic metabolic change patterns during grain development, it is interesting to investigate the co-regulated metabolites using correlation analysis based on the guilt-by-association principle, and to look for possible conserved metabolic pathways for future metabolic engineering. To this end, a pair-wise correlation analysis was performed by employing the Pearson’s product-moment correlation with 193 identified metabolites that were detected in more than 50% of grain samples.

At a threshold of absolute correlation value greater than 0.70 (|r-value| ≥ 0.70), there were 4642 pairs of positive correlations and 198 pairs of negative correlations (Supplementary Table S8). Most of the positive correlations were within primary metabolites and between primary metabolites and hydroxycinnamate derivatives (Supplementary Fig. S10), which is consistent with above mentioned metabolic change patterns in grains, where most primary metabolites and hydroxycinnamate derivatives showed similar change trends while secondary metabolites exhibited multi-directional change patterns. 85 and 71 of the 198 negative correlations were found to be connected with 2-LysoPC (14:0) and 1-LysoPC (14:0), respectively, probably because the fact that levels of these two lipids increased while most of the other metabolites decreased during grain development.

The highest correlation values were observed between metabolites with at least three different relations. The first relation was related to isomers, which had extremely high correlations. For example, the r-values between pipecolate I and pipecolate II, isoleucine and leucine, 1-LysoPE (18:2) and 2-LysoPE (18:2), 1-LysoPE (16:0) and 2-LysoPE (16:0), were all larger than 0.99 (P-value < 4.44E-16) (Table 1). The second relation was metabolites with substrate-product connection in the same metabolic pathway. For instance, the r-values of salicylic acid and salicylic acid 2-O-β-D-glucopyranoside, fumarate and malate, serine and glycine, phenylalanine and tyrosine were all higher than 0.98 (P-value < 4.44E-16) (Table 1). The third relation represented metabolites associated with the same substrate and same enzyme. For example, the r-value of isolecine and valine, both sharing the common substrate pyruvate and the same five enzyme systems in their biosynthesis pathway, was also extremely high (r-value = 0.987, P-value = 8.88E-16) (Table 1). Notably, the strongest association (r-value = 0.999, P-value < 4.44E-16) was found between momilactone A (formula C_20_H_26_O_3_) and momilactone A derivative (deduced formula C_20_H_29_NO_3_) (Table 1). Given that these two metabolites had the same retention time (RT = 13.87 min) and very similar MS/MS spectrum (Supplementary Fig. S11), we deduced that momilactone A derivative might be derived from momilactone A. In this sense, metabolite-metabolite correlation analysis could be useful for new pathway discovery.

## Discussion

Rice seed development undergoes profound changes in its metabolites, therefore, understanding of the molecular mechanisms underlying the rice seed development at metabolomic level is of fundamental importance not only for the basic understanding of rice biology but also for the applied rice breeding. In this study, taking the advantage of an established non-targeted metabolomics platform, we investigated into the kinetic changes of the metabolomes of rice grains at various developmental stages. Our results showed that the kinetics of metabolic changes in developing rice grain are similar among different cultivars although the metabolome of rice grain is cultivar and development stage dependent, which suggested a highly conserved metabolic regulation within rice seed development. In addition, our results also showed that some metabolic changes in developing grain of monocot rice share common characteristics with those of dicot Arabidopsis and tomato, while others show rice specific patterns.

Most of the metabolites, except for flavonoids, decreased from 7 DAF to 14 DAF in all four cultivars. Those metabolic change patterns of developing rice grains are consistent with previous proteomic and transcriptomic results^6^,^8^,^26^. Proteins related to central carbon metabolism (glycolysis and TCA cycle), amino acid metabolism, nucleotide metabolism and lipid metabolism were shown to be highly expressed from 2 to 8 DAF and down-regulated afterwards, although proteins involved in starch synthesis were highly expressed from 8–12 DAF and remained upregulated until seeds maturated^6^,^8^. The expression of genes coding the enzymes involved in carbohydrate metabolism, especially those in the TCA cycle, and amino acid metabolism were gradually decreased in embryo and endosperm from 7 DAF to 42 DAF^26^ (Supplementary Fig. S12). The incorporation of carbohydrates, amino acids and nucleotides into macromolecules, such as starch, storage proteins and DNA may contribute to the observed decrease of most detected metabolites in rice grains at the reserve accumulation stage as was previously hypothesized for Arabidopsis^22^. In addition, the accumulation of starch and storage proteins results in a greater than doubling of grain weight in 14 DAF grains as compared with those at 7 DAF^27^, which could be an alternative explanation for the observed decrease of the metabolite levels. Interestingly, the relative level of sucrose was rather stable from 7 DAF to 42 DAF in all four cultivars, indicating that carbon is replete during rice grain development and that the decrease of the metabolites in developing rice grains was not caused by the deficient of carbon sources from source tissues.

The grain weight doubled from 14 DAF to 28 DAF^27^, while the decreases of most amino acids, cofactors, nucleotides, hydroxycinnamate derivatives, carbohydrates and organic acids were much more than that could be explained simply by this weight increase. This discrepancy likely resulted from the grain physiological status, at 28 DAF, rice grain is in dormant and therefore no longer metabolically active, which is consistent with previous reports that there is a shift in grain from a highly active metabolic status to much less active dormant status^1^. Notably, asparagine, an ideal nitrogen transport and storage molecule^28^, was found to be considerably higher in rice grains at 28 DAF than that at 14 DAF in all four cultivars. In the rice grain asparagine is transported from the root through the xylem and from the leaves through the phloem and is thereafter rapidly catabolized into glutamate and other amino acids for seed development and germination^29^. Almost inactivated mobilization of asparagine in dormant rice grains was likely the cause of asparagine accumulation at 28 DAF.

Similar metabolic kinetics was also found in developing seeds of tomato and Arabidopsis^22^,^23^. The metabolome of tomato seeds at different developmental stages were different with the largest differences were found between the seed at the earliest stage (8 DAF) and that at the latest stage (45 DAF). Sugars, amino acids and organic acids decreased significantly at 45 DAF as compared with those at 8 DAF. The degree of decrease for many metabolites, such as fructose, glucose, GABA, glutamate, glutamine, 5-oxoproline, valine, citrate and linolenate, between the last developmental stage and the earliest stage in rice grain and tomato seeds were very similar. The metabolome of Arabidopsis seeds at different developmental stages differed as well, with the progression of Arabidopsis seeds from 10 DAF to 14 and 17 DAF being associated with major decreases in the levels of most amino acids, sugars, polyols, and organic acids. Abovementioned results indicated that there is a conserved metabolic shift in plant seeds, a typical sink tissue, from simple soluble metabolites to complex macromolecules, which occurred mainly at the late organogenesis stage and early seed maturation stage. This appears to be a unique metabolic feature of sink tissues since it is not observed in source tissues, such as in Arabidopsis leaves^30^.

The major difference in metabolite shifts between Arabidopsis seeds and rice grains is apparent at the desiccation stage. Desiccating Arabidopsis seeds are associated with a general increase in most amino acids, sugars (including raffinose), polyols, organic acids (with the exception of TCA intermediates), fatty acids and fatty acid-related compounds^22^, while desiccating rice grains are associated with significant decreases of these metabolites (except asparagine). In addition, levels of palmitate and oleate increased at reserve accumulation stage and dropped afterwards in developing Arabidopsis seeds, but constitutively decreased from the beginning in developing rice grains. These observed metabolic differences between developing grains of monocot and dicot plants indicated possible different regulatory machinery operating in the process of seed desiccation. There is striking difference in the seed structure between monocot and dicot plants, the major component of mature rice seed is lifeless endosperm while that of Arabidopsis mature seed is *vivo* embryo, which might be a causing factor for the observed metabolic differences.

Mature seeds of *Japonica* and *indica* rice differ profoundly in their contents and molecular fine structure of amylose and amylopectin, eating and cooking quality and metabolite levels^20^,^31^,^32^,^33^. These morphological and physiological differences are likely associated with the distinguishable metabolic abundance of *japonica* and *indica* developing grains. Although we here provide direct evidence that the largest metabolic difference between *japonica* and *indica* developing grains was found at the reserve accumulation stage especially at 10 DAF, the molecular mechanisms underlying the metabolic variation between these two subspecies merits future investigations.

Metabolite-metabolite correlation analysis can be used to uncover the relationships amongst metabolite. In this study, metabolite-metabolite correlation analysis revealed that the levels of primary metabolites in developing rice grains was positively associated, which is similar to our previous findings in various rice mature seeds^20^. Notably, the r-values observed in developing grains in this study were higher than those found in mature seeds. This could result from the fact that almost all the primary metabolites were decreased from 7 DAF to 28 DAF in all four cultivars while the metabolic changes in grains from 28 DAF to 42 DAF were more cultivar specific as described above. It is noteworthy that metabolite pairs with the top r-values have direct connection in specific metabolic pathways, which would facilitate the metabolite annotation/identification and new pathway discovery, when combined with accurate mass of the metabolite and MS/MS spectrum information.

It is worthy to note that in this study the metabolomics platform we used only identified 214 metabolites from detected 3011 metabolic features. First, the limited number of MS/MS spectrums in our in-house metabolite library and public database restricted the number of metabolites being identified. Second, the nature of highly diverse properties and abundances of plant metabolites rendered it impossible to detect all of them using one extraction method or single platform^34^. In this study, we used methanol based extraction solution to extract a broad range of metabolites, and employed LC-MS/MS and GC-MS to identify as many metabolites as possible for a global purpose. Therefore, this approach is not the best way to identify targeted metabolites. In the future, optimized extraction methods and/or detecting methods for targeted metabolites, such as acyl CoAs^35^, lipids^36^,^37^ and volatiles^38^,^39^,^40^,^41^, should be combined with current non-targeted approach, to better understand the whole metabolomic changes occurring in developing rice grains.

In summary, our study not only corroborates published transcriptomic and proteomic data but also brought new insights concerning the developing rice grain metabolomes with the observed metabolic kinetics corresponding well to the physiological processes occurring in the developing rice grains. Whilst some of these metabolic changes share common characteristics with reported developing seeds of Arabidopsis and tomato, others show rice specific patterns. As such this study demonstrates both conserved and diverse elements of the metabolic regulatory system of plant seeds. This observation merits further study both to explore fundamental questions regarding the evolution of seed metabolic capabilities as well as their potential applications in crop improvement.

## Methods

### Materials

Rice plants were planted in a paddy filed in Minghang (31.03°N, 121.45°E), Shanghai, during the summer season in 2013. Four biological replications of rice seeds at 7, 10, 14, 28 and 42 DAF were collected, immediately frozen with liquid nitrogen, lyophilized for 48 hours and stored at −80 °C until metabolomics analysis.

### Metabolite Profiling

Samples were grounded into fine powder, and methanol extracts from 20 mg sample were then analyzed by GC-MS and UHPLC-MS (positive and negative mode). The detailed information concerning GC-MS analysis can be found in published paper^22^,^42^, with data acquisition and processing being performed with TagFinder software^43^. Raw data of GC-MS can be downloaded from Metabolights website (http://www.ebi.ac.uk/metabolights/) with the study identifier MTBLS288.

LC analysis was performed on the Aglient 1290 Infinity II LC^TM^ system. Samples were injected into an Agilent Eclipse-plus C18 column (150 × 3.0 mm i.d., 1.8 μm), and the column temperature was set at 40 °C. The mobile phase consisted of A (0.1% formic acid in water) and B (100% acetonitrile). The gradient conditions of the mobile phase as follows: 0 min, 98.0% A; 1.0 min, 98% A; 5.0 min, 60% A; 12.0 min, 30% A; 15.0 min, 5% A; 20.0 min, 5% A. The flow rate was 0.40 ml/min. The sample injection volume was 1.00 μl. MS detection was performed on a Agilent 6550 iFunnel/Q-TOF mass spectrometer with Agilent Jet-Stream source. Full scan mass spectra were recorded through a range of 50–1000 m/z with scan rate at 2 spectra/sec. The ESI source was operated in positive and negative ionization modes with a capillary voltage of 3.5kV for both modes, nozzle voltage at 250V (+) and 1500V (-), fragmentor voltage of 380V, nebulizer was 25psi, sheath gas and dry gas were set at the flow rate of 12L/min and 16L/min, respectively, and CID voltage applied with 10V, 20V and 40V. Metabolites were annotated by searching Personal Compound Database and Library (PCD/PCDL), Metlin database^44^, Massbank database^45^ and literatures^18^,^46^,^47^. Data acquisition, review, alignment and normalization to Quality Control (QC) sample were performed with the softwares of MassHunter Acquisition 6.0, MassHunter Qualitative 6.0, Mass Profinder 6.0 and Mass Profiler Professional 13.0 (MPP 13.0), respectively. Raw data of UHPLC-MS can be downloaded from Metabolights website with the study identifier MTBLS286 and MTBLS287 for positive and negative ionization mode, respectively.

### Data Analysis

The sample weight and non-normalized peak areas are available in Supplementary Table S9. For data normalization, peak areas were divided by the sample weight and the median value of each metabolite. The missing values of a giving metabolite were imputed with the detected minimum value for statistical analysis, assuming that they were below the limits of instrument detection sensitivity. The final statistics matrix with normalized data for the following statistical analysis are available in Supplementary Table S10. Metabolic differences between rice grains at different stages were determined using the *t*-test algorithm embedded in Excel 2013 and deemed significant at level of 5%. Principle Component Analysis was performed with SIMCA-P version 11.0. Two-way ANOVA and ASCA were performed with MetATT^48^ using “Pareto Scaling” for data normalization. Two-way ANOVA type used is “within subjects ANOVA”, significance threshold is defined as the corrected P-value < 0.05 and False Discovery Rate is chosen for multiple testing correction. ASCA was performed with default parameters. Metabolite-metabolite correlations were analyzed by using Pearson correlation method in R software. Metabolic pathways and the graphical presentation of metabolite-metabolite correlations were presented with Cytoscape version 2.8.3. The heatmaps of metabolite ratios were visualized with MultiExperiment Viewer (MeV) version 4.8.

## Additional Information

**How to cite this article**: Hu, C. *et al.* Identification of Conserved and Diverse Metabolic Shifts during Rice Grain Development. *Sci. Rep.* **6**, 20942; doi: 10.1038/srep20942 (2016).

## Supplementary Material

Supplementary Information

Supplementary Dataset 1

## Figures and Tables

**Table 1:** The top 10 metabolite pairs with the strongest metabolite-metabolite correlations in developing rice grains

Metabolite 1	Metabolite 2	Relationship	R-value	P-value
Momilactone A	Momilactone A derivative	2′	0.999	<4.44E-16
Pipecolate I	Pipecolate II	1	0.998	<4.44E-16
Salicilate	Salicylic acid 2-O-β-D-glucopyranoside	2	0.997	<4.44E-16
Fumarate	Malate	2	0.996	<4.44E-16
Isoleucine	Leucine	1	0.994	<4.44E-16
1-LysoPE(18:2)	2-LysoPE(18:2)	1	0.993	<4.44E-16
1-LysoPE(16:0)	2-LysoPE(16:0)	1	0.992	<4.44E-16
Glycine	Serine	2	0.988	4.44E-16
Phenylalanine	Tyrosine	2	0.988	4.44E-16
Isoleucine	Valine	3	0.987	8.88E-16

“Relationship 1” indicates that Metabolite 1 and Metabolite 2 are isomers with similar MS/MS spectrum; “Relationship 2” indicates that Metabolite 1 and Metabolite 2 are of substrate-product relationship in the same metabolic pathway; “Relationship 2′” indicates that Metabolite 1 and Metabolite 2 are deduced to be of substrate-product relationship; “Relationship 3” indicates that Metabolite 1 and Metabolite 2 are metabolites with the same substrate and the same catalytic enzymes.
