# Supplementary material for: Identification of Conserved and Diverse Metabolic Shifts during Rice Grain Development
Source: Sci Rep. 2016 Feb 10;6:20942. doi: 10.1038/srep20942 (PMC4748235; doi:10.1038/srep20942)
Supplement: Supplementary Information [file srep20942-s1.pdf]

Supplementary Information

Supplementary Figures

Supplementary Figure S1 | Scree plots of each factor.

Supplementary Figure S2 | Score plots associated with cultivar and the interaction based on PC1 of the corresponding submodels..

Supplementary Figure S3 | ASCA selection of important metabolites associated with cultivar and interaction..

Supplementary Figure S4 | Variations of representative metabolites well-modeled by developmental stage.

Supplementary Figure S5 | Heat map of metabolite changes in rice grains at reserve accumulation stage (7, 10 and 14 DAF).

Supplementary Figure S6 | Heat map of metabolite changes in rice grains at desiccation stage (28 DAF vs 14 DAF).

Supplementary Figure S7 | Principal component analysis of the rice grain metabolome at 28 DAF and 42 DAF.

Supplementary Figure S8 | Heat map of metabolite changes in rice grains at dormancy stage (42 DAF vs 28 DAF).

Supplementary Figure S9 | Heat map of metabolic difference in rice grains between two japonica cultivars and two indica cultivars at different developmental stages.

Supplementary Figure S10 | Heatmap of metabolite-metabolite correlation and significance in developing rice grains.

Supplementary Figure S11 | MS/MS spectrum of momilactone A and momilactone A derivate.

Supplementary Figure S12 | Supplementary Figure S12 Gene expression patterns during rice seed development.

Supplementary Tables

Supplementary Table S1. List of metabolites identified in developing rice grain.

Supplementary Table S2. Results of two-way ANOVA.

Supplementary Table S3. List of well-modeled metabolites

Supplementary Table S4. Changes in the levels of metabolites in rice grains at reserve accumulation stage. Values of 10 and 14 DAF were normalized to the mean response calculated for 7 DAF, which was given the value of 1.

Supplementary Table S5. Changes in the levels of metabolites in rice grains at seed desiccation stage

Supplementary Table S6. Changes in the levels of metabolites in rice grains at seed dormancy stage.

Supplementary Table S7. Changes in the levels of metabolites in rice grains at the same developmental stage between two japonica and two indica cultivars.

Supplementary Table S8. Data of metabolite-metabolite correlations analysis.

Supplementary Table S9. The sample weight and original peak areas extracted from chromatogram by using MassProfinder.

Supplementary Table S10. The final statistics matrix with normalized data

Supplementary Figures

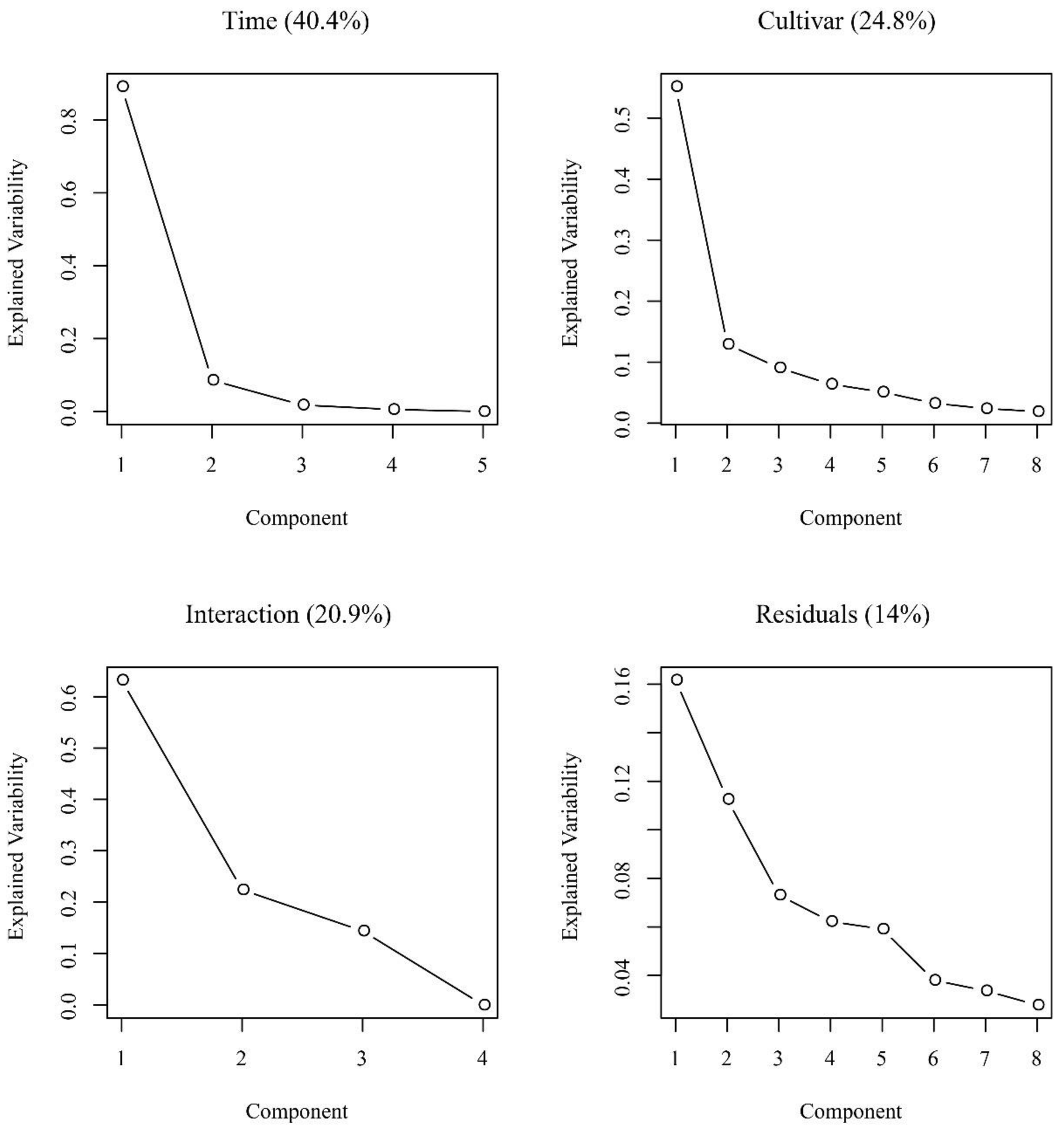

Supplementary Figure S1 | Scree plots of each factor.

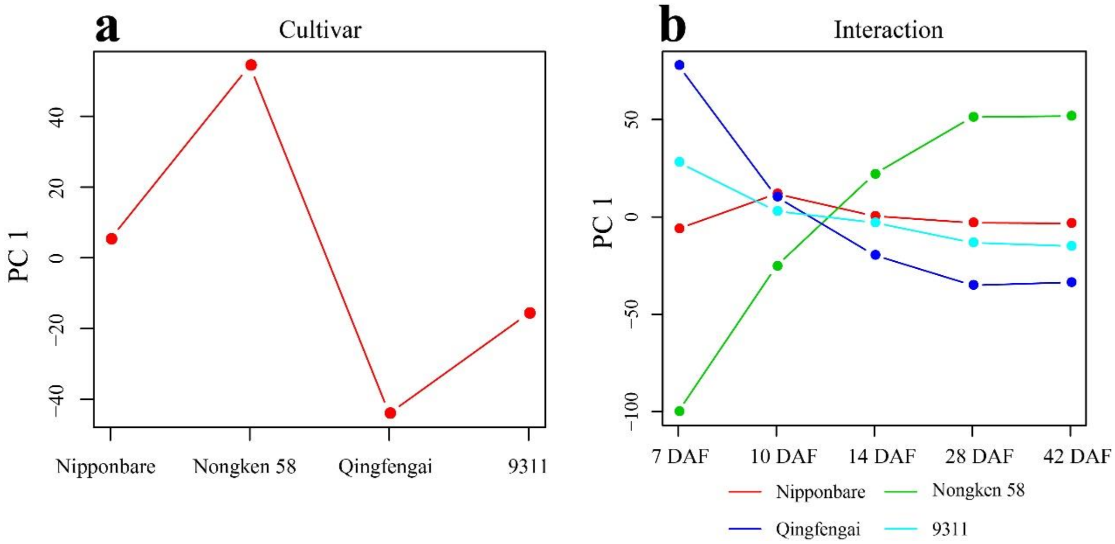

Supplementary Figure S2 | Score plots associated with cultivar and the interaction based on PC1 of the corresponding submodels.

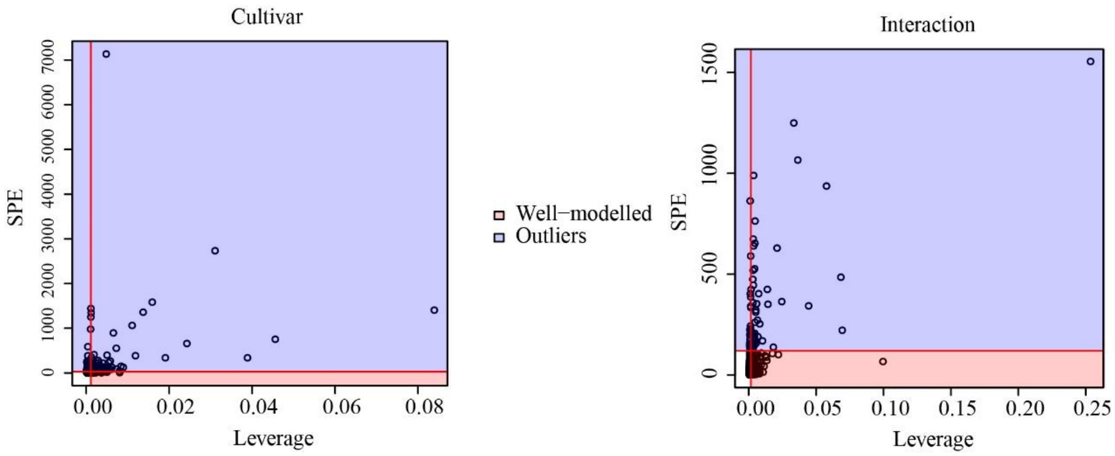

Supplementary Figure S3 | ASCA selection of important metabolites associated with cultivar and interaction.

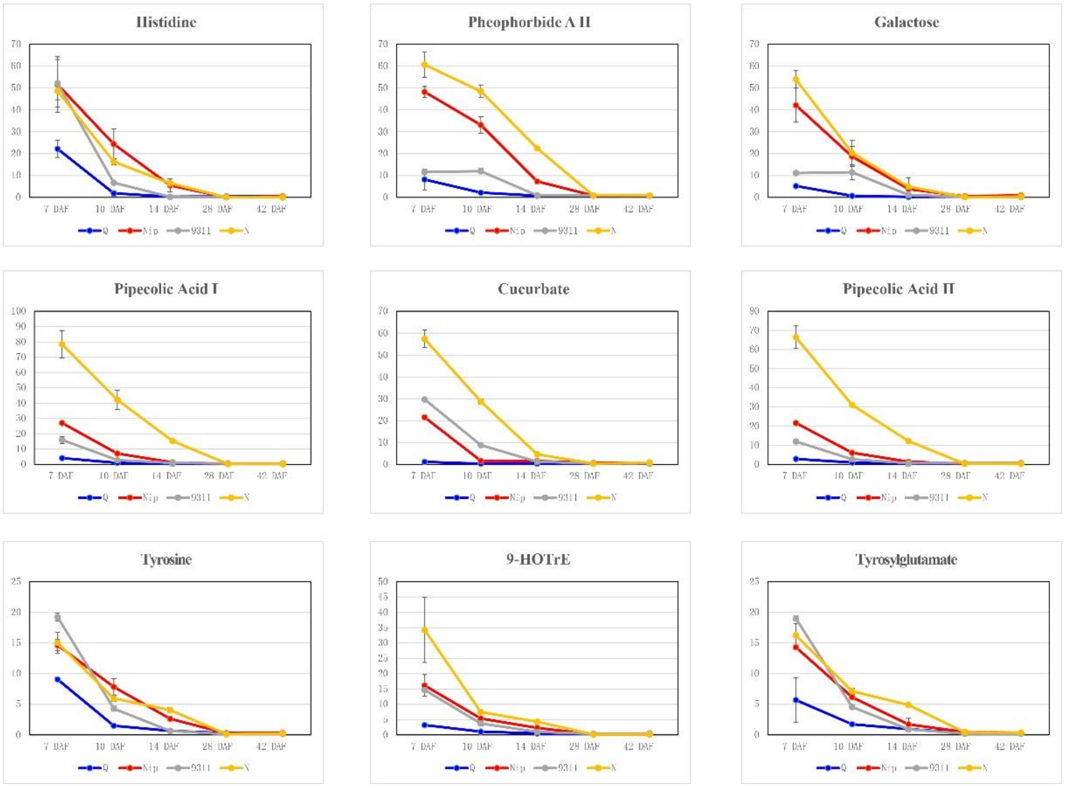

Supplementary Figure S4 | Variations of representative metabolites well-modeled by developmental stage.

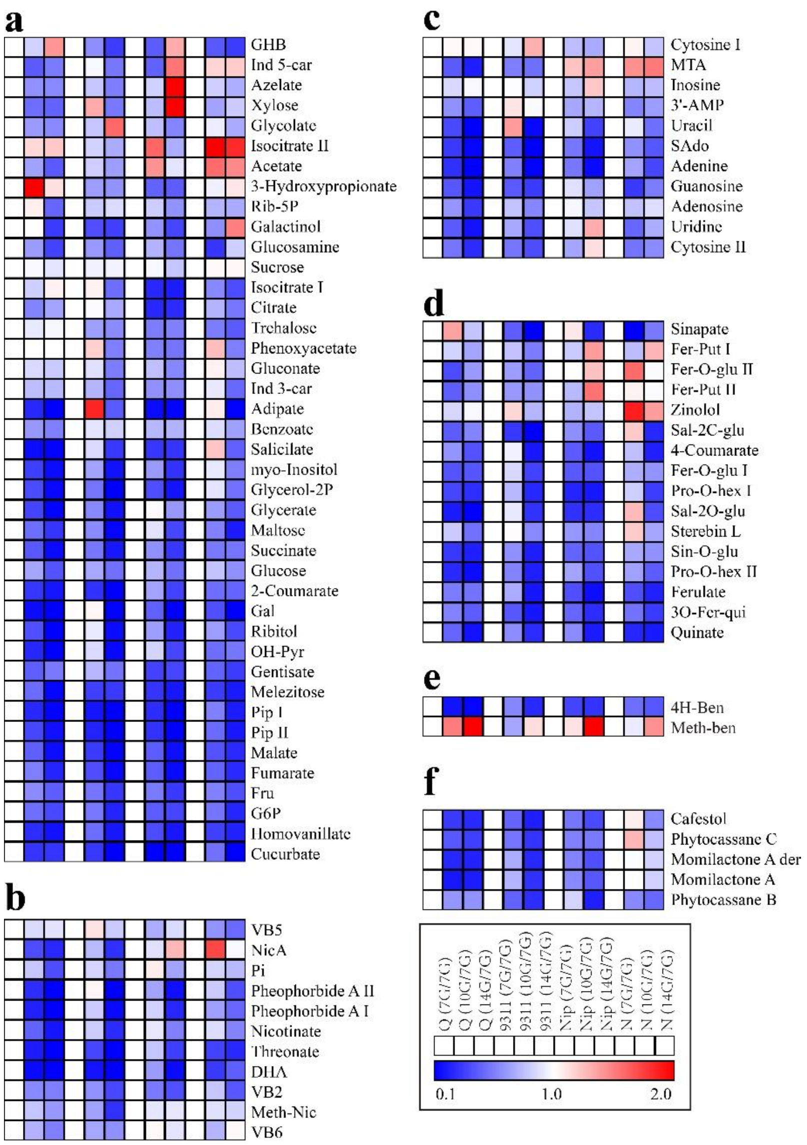

Supplementary Figure S5 | Heat map of metabolite changes in rice grains at reserve accumulation stage (7, 10 and 14 DAF). (a) carbohydrates and organic acids. (b) cofactors. (c) nucleotides. (d) hydroxycinnamate derivatives and their derivatives . (e) phenylpropanoids. (f) terpenes and diterpenoid phytoalexins. Q, Nip and N are shorted for three rice cultivars, Qingfengai, Nipponbare and Nongken 58, respectively. Ratios of fold changes are given by shades of red or blue colors according to the scale bar. Data represent mean values of four biological replicates for each cultivar and time point. Statistical analysis was performed using t-test (Supplementary Table S4). For full metabolite names, refer to Supplementary Table S1.

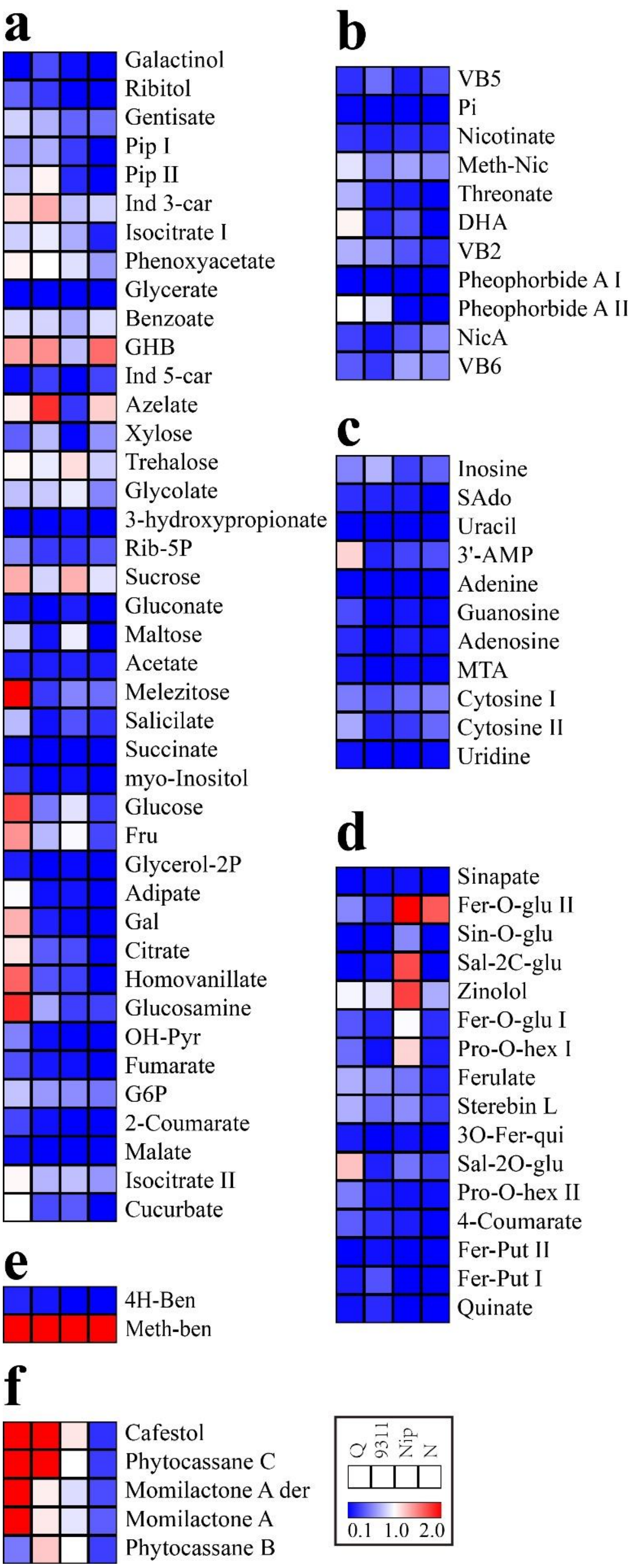

Supplementary Figure S6 | Heat map of metabolite changes in rice grains at desiccation stage (28 DAF vs 14 DAF). (a) carbohydrates and organic acids. (b)

cofactors. (c) nucleotides. (d) hydroxycinnamate derivatives and their derivatives . (e) phenylpropanoids. (f) terpenes and diterpenoid phytoalexins. Q, Nip and N are shorted for three rice cultivars, Qingfengai, Nipponbare and Nongken 58, respectively. Ratios of fold changes are given by shades of red or blue colors according to the scale bar. Data represent mean values of four biological replicates for each cultivar and time point. Statistical analysis was performed using t-test (Supplementary Table S5). For full metabolite names, refer to Supplementary Table S1.

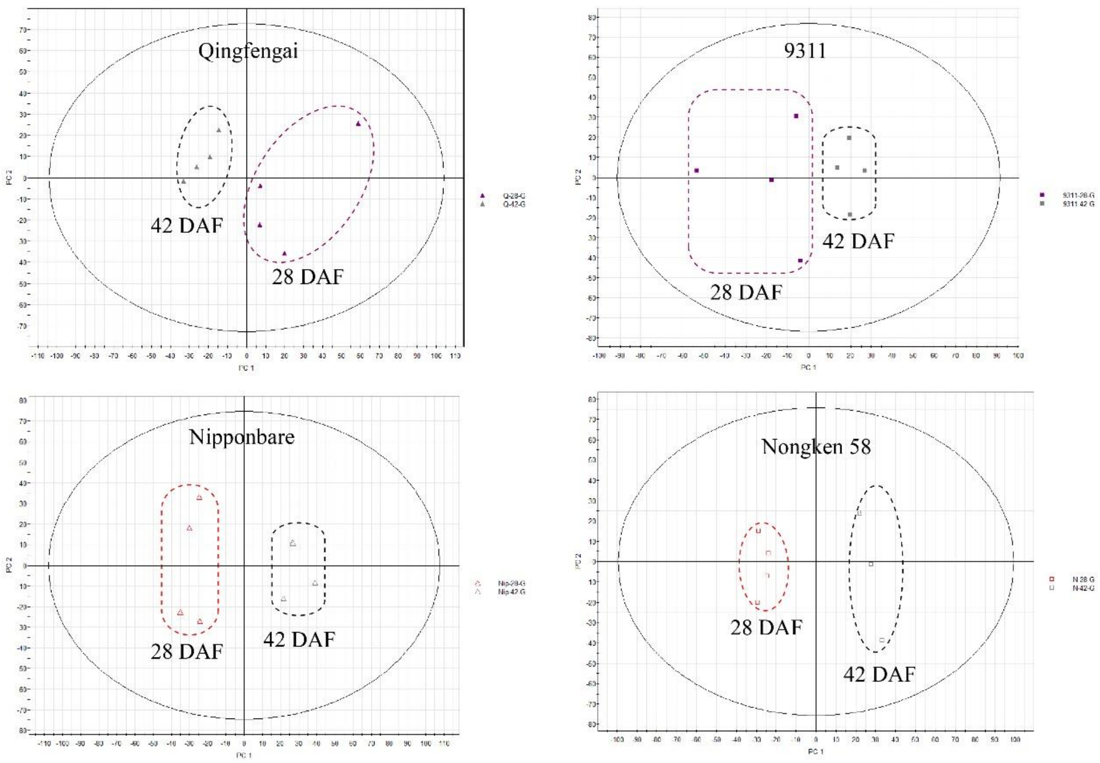

Figure S7 | Principal component analysis of the rice grain metabolome at 28 DAF and 42 DAF.

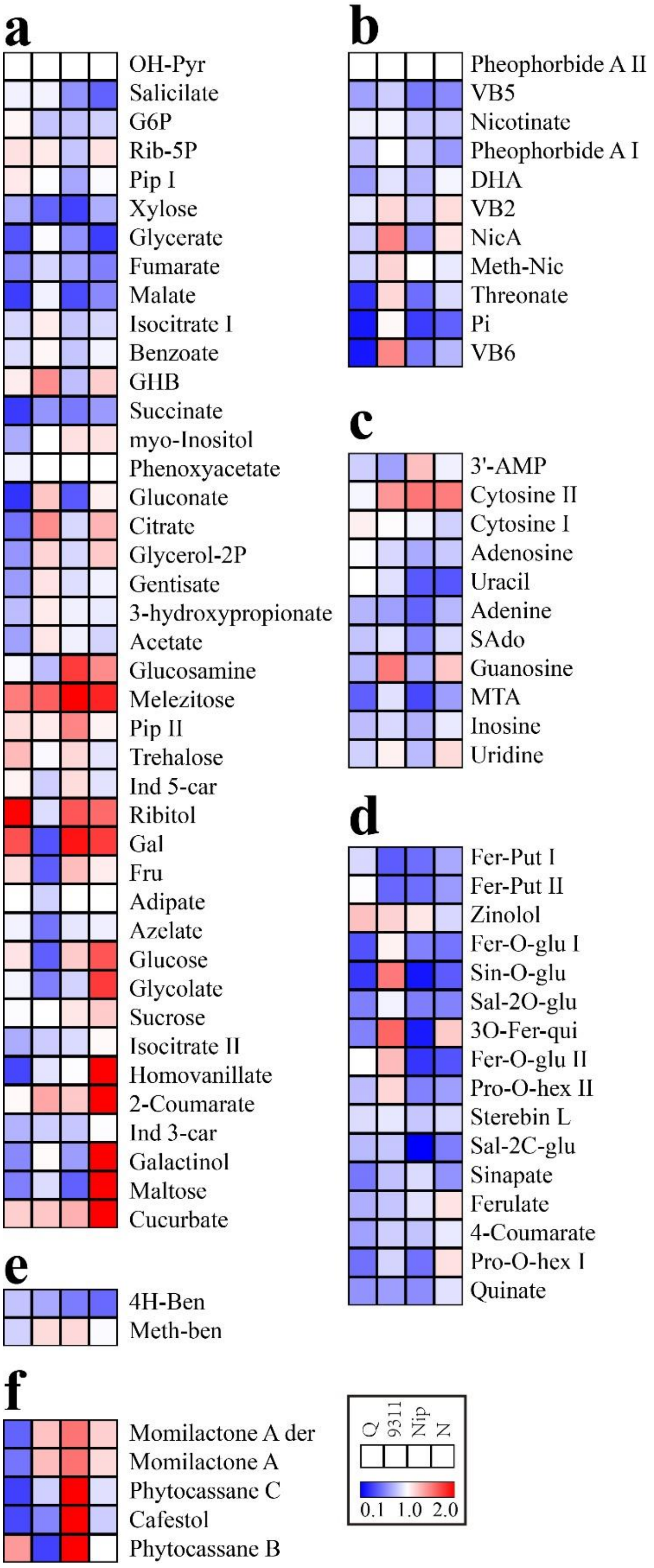

Supplementary Figure S8 | Heat map of metabolite changes in rice grains at dormancy stage (42 DAF vs 28 DAF). (a) carbohydrates and organic acids. (b) cofactors. (c) nucleotides. (d) hydroxycinnamate derivatives and their derivatives . (e) phenylpropanoids. (f) terpenes and diterpenoid phytoalexins. Q, Nip and N are shorted

for three rice cultivars, Qingfengai, Nipponbare and Nongken 58, respectively. Ratios of fold changes are given by shades of red or blue colors according to the scale bar. Data represent mean values of four biological replicates for each cultivar and time point. Statistical analysis was performed using t-test (Supplementary Table S6). For full metabolite names, refer to Supplementary Table S1.

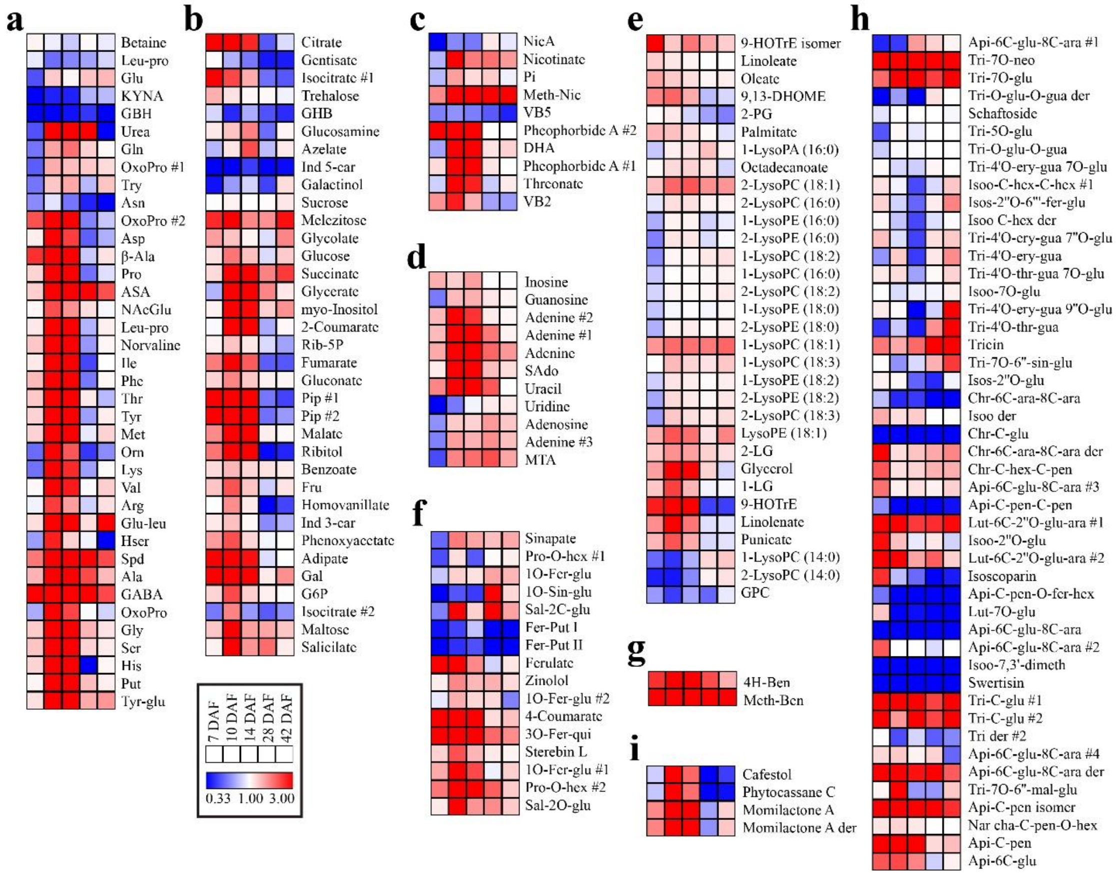

Supplementary Figure S9 | Heat map of metabolic difference in rice grains between two *japonica* cultivars and two *indica* cultivars at different developmental stages. Log_2 ratios of fold changes are given by shades of red or blue colors according to the scale bar. (a) amino acids and dipeptides. (b) carbohydrates and organic acids. (c) cofactors. (d) nucleotides. (e) lipids. (f) hydroxycinnamate derivatives and their derivatives. (g) phenylpropanoids. (h) flavonoids. (i) terpenes and diterpenoid phytoalexins. Statistical analysis of metabolic difference between *japonica* and *indica* rice was performed using t-test (Supplementary Table S7). For full metabolite names, refer to Supplementary Table S1.

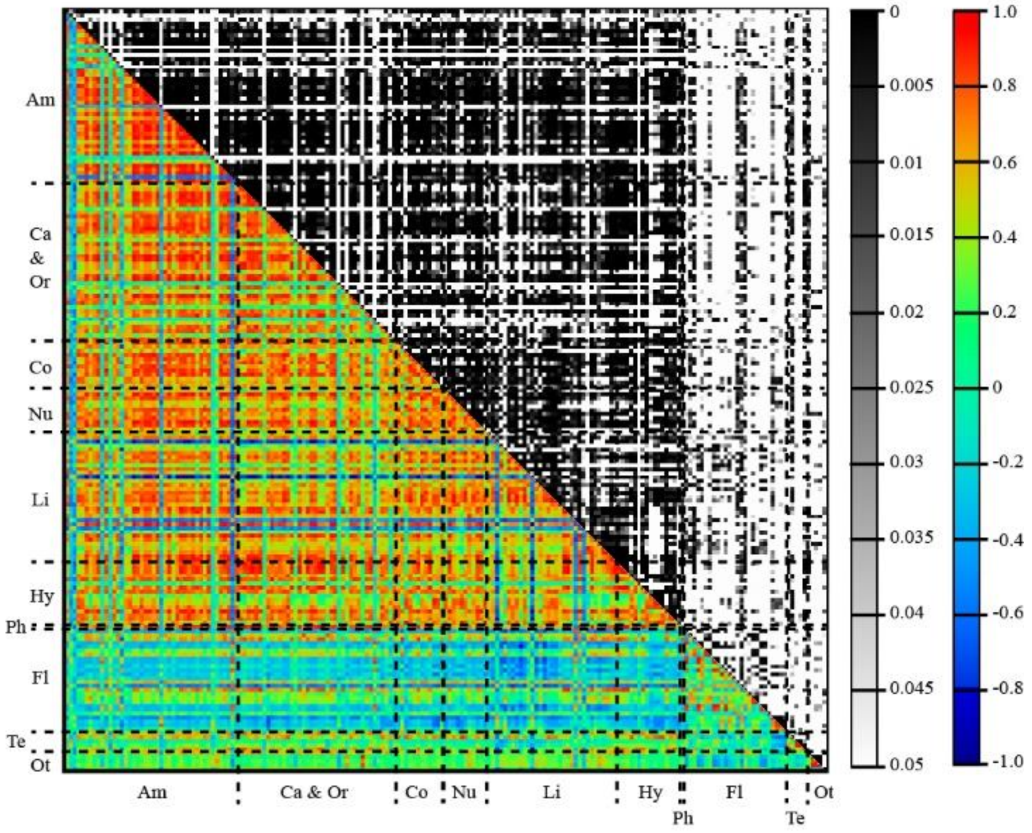

Supplementary Figure S10 | Heatmap of metabolite-metabolite correlation and significance in developing rice grains. In the colored area, rectangles represent Pearson correlation coefficient (r) values of metabolite pairs according to the correlation color key. In the black and white area, rectangles represent the respective p-values according to the significance color key. Abbreviations of metabolite classes are as follows: Am: amino acids and dipeptides; Ca & Or, carbohydrates and organic acids; Co, cofactors; Nu, nucleotides; Li, lipids; Hy, hydroxycinnamate derivatives; Ph, phenylpropanoids; Fl, flavonoids; Te, terpenes and diterpenoid phytoalexins; Ot, others. Detailed information of metabolite-metabolite correlation can be foud in Supplementary Table S8.

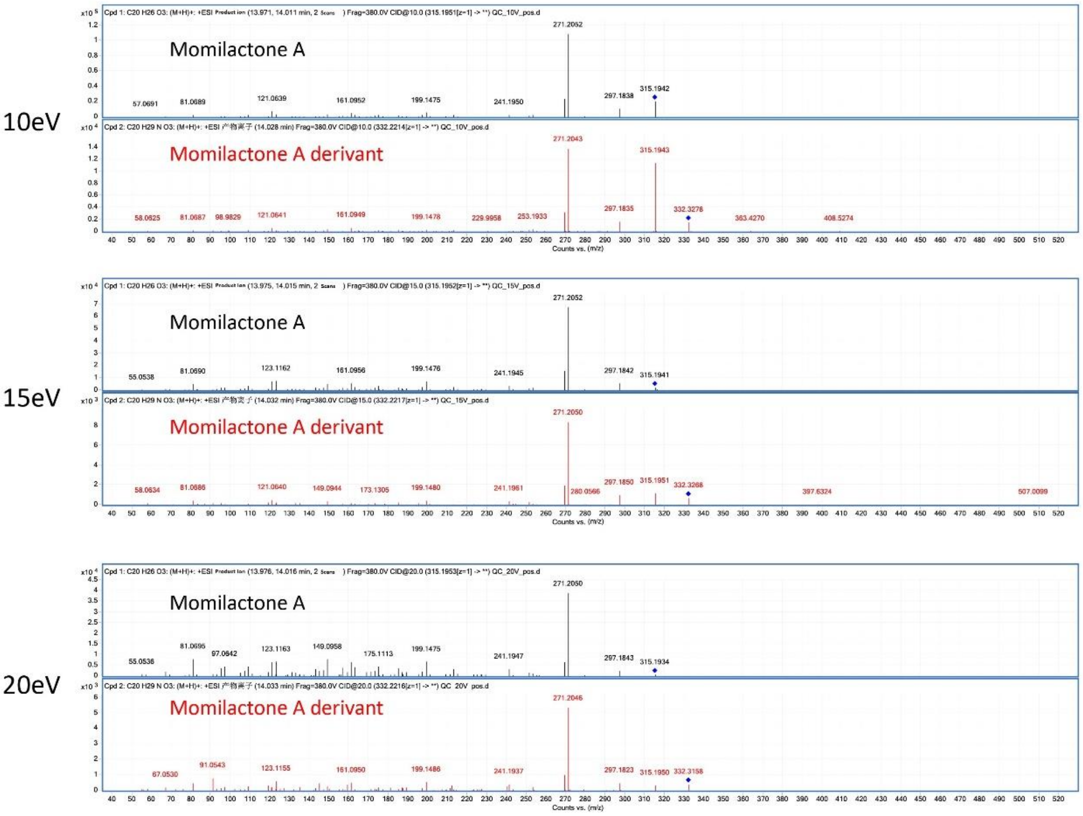

Supplementary Figure S11 | MS/MS spectrum of momilactone A and momilactone A derivate.
